# The impact of glucagon-like peptide-1 receptor agonists in the patients undergoing anesthesia or sedation: systematic review and meta-analysis

**DOI:** 10.1186/s13741-024-00439-y

**Published:** 2024-07-22

**Authors:** Tatiana S. do Nascimento, Rodrigo O. L. Pereira, Eduardo Maia, Tetsu Ohnuma, Mariana G. da Costa, Eric Slawka, Carlos Galhardo, Vijay Krishnamoorthy

**Affiliations:** 1 Department of Anesthesiology, Cardoso Fontes Federal Hospital, Av. Menezes Cortes, Rio de Janeiro, RJ 3245 Brazil; 2 Division of Cardiothoracic Anesthesiology, Department of Anesthesiology, Duke University Hospital, Durham, NC USA; 3 School of Medicine, Federal University of Minas Gerais, Belo Horizonte, MG Brazil; 4 Division of Critical Care, Department of Anesthesiology, Duke University Hospital, Durham, NC USA; 5 Department of Anesthesiology, University Medical Center of Groningen, Groningen, The Netherlands; 6 School of Medicine, Federal University of Rio de Janeiro, Rio de Janeiro, RJ Brazil; 7 Department of Anesthesiology, McMaster University & DeGroot School of Medicine, Hamilton, ON Canada

## Abstract

**Background:**

Glucagon-like peptide-1 agonist receptors (GLP-1RAs), medications used for glycemic control and weight loss, are increasing worldwide. In the perioperative period, the major concern related to GLP-1RA is gastric emptying delay and risk of aspiration. This meta-analysis and systematic review compared the risks and benefits of using GLP-1 agonist receptors and control in surgical and nonsurgical procedures under anesthesia or sedation.

**Methods:**

We systematically searched MEDLINE, Embase, and Cochrane for randomized controlled trials and observational studies involving patients > 18 years undergoing elective surgeries or procedures. Outcomes of interest were pre-procedural gastrointestinal (GI) symptoms, residual gastric content assessed by endoscopy, pulmonary aspiration during anesthesia/sedation, perioperative glycemic control, postoperative inotropic support, nausea/vomiting (PONV), atrial fibrillation, and 30-day mortality rate. We used a random effects model, with odds ratio and mean difference computed for binary and continuous outcomes, respectively.

**Results:**

Fourteen randomized and observational studies with 2143 adult patients undergoing elective surgeries and procedures were included. GLP-1RA resulted in increased pre-procedural GI symptoms (*OR* 7.66; 95% *CI* 3.42, 17.17; *p* < 0.00001; *I*^2^ = 0%) and elevated residual gastric content (*OR* 6.08; 95% *CI* 2.86, 12.94; *p* < 0.00001; *I*^2^ = 0%). GLP-1RA resulted in lower glycemic levels (*MD* − 0.73; 95% *CI* − 1.13, − 0.33; *p* = 0.0003; *I*^2^ = 90%) and lower rate of rescue insulin administration (*OR* 0.39; 95% *CI* 0.23, 0.68 *p* = 0.0009; *I*^2^ = 35%). There was no significant difference in rate of perioperative hypoglycemia (*OR* 0.60; 95% *CI* 0.29, 1.24; *p* = 0.17; *I*^2^ = 0%), hyperglycemia (*OR* 0.89; 95% *CI* 0.59, 1.34; *p* = 0.58; *I*^2^ = 38%), need for postoperative inotropic support (*OR* 0.57; 95% *CI* 0.33, 1.01; *p* = 0.05; *I*^2^ = 0%), atrial fibrillation (*OR* 1.02; 95% *CI* 0.52, 2.01; *p* = 0.95; *I*^2^ = 16%), rate of PONV (*OR* 1.35; 95% *CI* 0.82, 2.21; *p* = 0.24; *I*^2^ = 0%), and 30-day mortality rate (*OR* 0.54; 95% *CI* 0.14, 2.05; *p* = 0.25; *I*^2^ = 0%).

**Conclusion:**

Compared to control**,** pre-procedural GLP-1RA increased the rate of GI symptoms and the risk of elevated residual gastric content despite adherence to fasting guidelines. GLP-1RA improved glycemic control and decreased the rate of rescue insulin administration. There was no significant difference in the rates of perioperative hypo or hyperglycemia, postoperative inotropic support, PONV, atrial fibrillation, and 30-day mortality.

**Supplementary Information:**

The online version contains supplementary material available at 10.1186/s13741-024-00439-y.

## Key points summary

• Question: What are the benefits and risks of using GLP-1RAs in patients undergoing anesthesia or sedation?

• Finding: This meta-analysis demonstrates that perioperative GLP-1RAs increased the rate of GI symptoms and the risk of elevated residual gastric content, improved glycemic control, and did not change the rates of hypo or hyperglycemia, postoperative inotropic support, PONV, atrial fibrillation, and 30-day mortality.

• Meaning: Despite improving glycemic control, providers must be aware of the potential risk of anesthesia-related gastrointestinal dysfunction and pulmonary aspiration associated with using GLP-1RA.

**Supplementary Information:**

The online version contains supplementary material available at 10.1186/s13741-024-00439-y.

## Introduction

Glucagon-like peptide-1 receptor agonists (GLP-1RA) are gut-derived incretin-mimetic hormones that stimulate insulin secretion, suppress glucagon release, increase satiety, slow gastric emptying, and inhibit small bowel motility (Camilleri and Lupianez-Merly 2023; Horowitz et al. 2017). GLP-1RA also decreases the rate of cardiovascular events and slows renal function decline in diabetic patients (Granata et al. 2022; Holman et al. 2017). For these reasons, GLP-1RA are increasingly popular options for glycemic control and weight management in patients with obesity and type 2 diabetes (Camilleri and Lupianez-Merly 2023; Jones et al. 2023).

As the prescription of GLP-1RA increases, it becomes essential for medical providers to understand these medications’ pharmacology and physiologic implications, including the potential benefits and risks they may convey for the surgical patient (Joshi et al. 2023; Xu et al. 2022). One concerning effect of GLP-1RA is delaying gastric emptying, leading to increased gastric volumes, and putting patients at risk for aspiration during anesthesia and/or sedation (Beam 2023; Kaneko et al. 2018). On the other hand, the perioperative administration of GLP-1RA may facilitate glycemic control, minimizing glucose level variability and decreasing insulin requirements (Beam 2023; Kaneko et al. 2018; Hulst et al. 2019).

A previous meta-analysis revealed that GLP-1RA improves glycemic control after coronary artery bypass surgery (Watkins et al. 2023). However, the authors did not assess the impact of GLP-1RA on pre-procedural residual gastric volume, which is important for perioperative care. Furthermore, the impact of GLP-1RA use in noncardiac surgery is largely unknown.

To address these gaps, this meta-analysis and systematic review investigates the benefits and risks of using GLP-1RA in surgical and nonsurgical procedures under anesthesia or sedation compared to the standard of care.

## Material and methods

This systematic review and meta-analysis was conducted and reported based on the Preferred Reporting Items for Systematic Reviews and Meta-analyses (*PRISMA*) and the *Cochrane Handbook for Systematic Reviews of Intervention* guidelines (Higgins 2022). The predefined protocol of the present study was registered in the International Prospective Register of Systematic Reviews (*PROSPERO*: identifier CRD42023469511).

### Eligibility criteria

Inclusion in this meta-analysis was restricted to studies that met all the following eligibility criteria: (1) randomized trials and nonrandomized studies; (2) comparing GLP-1RA to insulin or placebo; (3) comparing GLP-1RA to no GLP-1RA; (4) enrolling patients who underwent elective surgeries or procedures, including endoscopies; and (5) studies were included only if they reported any of the clinical outcomes of interest. We excluded studies with (1) patients younger than 18 years old, (2) in vitro, (3) in animals, (4) trial protocols, and (5) abstracts without peer-reviewed publications of a manuscript.

The primary outcomes were pre-procedural gastrointestinal (GI) symptoms (nausea, vomiting, dyspepsia, abdominal distension), increased residual gastric content (RGC), and pulmonary aspiration related to anesthesia/sedation.

The secondary outcomes were glycemic control (hypoglycemia, hyperglycemia, mean blood glucose levels, rate of rescue insulin administration), rate of postoperative inotropic support (use of milrinone, epinephrine, or dobutamine), 30-day atrial fibrillation, 30-day mortality, and postoperative nausea/vomiting.

### Search strategy

The research reported in this systematic review and meta-analysis followed PRISMA guidelines. We systematically searched using MEDLINE, Embase, and Cochrane electronic databases. We also searched for references in the selected articles. The final search was performed on January 14, 2024, using the following medical subject heading terms: Semaglutide, Ozempic, Wegovy, Rybelsus, taspoglutide, liraglutide, Victoza, Saxenda, dulaglutide, Trulicity, albiglutide, Eperzan, exenatide, exendin, Byetta, lixisenatide, Lyxumia, tirzepatide, glucagon-like peptide-1 receptor agonists, GLP-1, incretin, endoscopy, esophagogastroduodenoscopy, anesthesia, and perioperative.

### Study selection

Two independent reviewers (T. S. N., R. O. L. P.) selected eligible studies based on the inclusion and exclusion criteria, and a cross-validation was performed. After removing the duplicates and retracting the studies, all were pooled and selected for inclusion in the meta-analysis based on their titles or abstracts. Finally, the remaining articles were read in full to assess eligibility. Any disagreement between the two reviewers was resolved by discussing it with a third reviewer (E. M.).

### Data extraction

After finishing the study selection, the final studies (randomized and non-randomized) underwent data extraction to summarize the following variables: author, publication year, type of study, population, intervention, control, type of surgery and procedure, and the result of outcomes of interest. When continuous data were reported as a median and interquartile range, we used the Wan et al. method to convert them to mean and standard deviation (Luo et al. 2018).

### Quality assessment

The revised Cochrane risk-of-bias tool for randomized trial 2 (*RoB-2*) was used for randomized studies. It consists of five categories: (1) bias arising from the randomized process, (2) bias due to deviations from intended interventions, (3) bias caused by missing outcome data, (4) bias in the measure of outcome, and (5) bias in the selection of the reported result. This risk of bias was categorized as low risk, some concerns, or high risk (Sterne et al. 2019).

The revised Cochrane risk-of-bias tool for the nonrandomized trial I (*ROBINS-I*) was used for nonrandomized studies. It consists of three stages: stage 1 — planning, stage 2 — risk-of-bias assessment for specific results, and stage 3 — overall risk-of-bias assessment. This risk of bias was categorized as low risk, moderate risk, serious risk, critical risk, and no information (Sterne et al. 2016). Two independent authors (T. S. N., E. M.) reviewed the risk of assessment bias, and any disagreements were decided among the authors.

#### Certainty of evidence assessment

*The Grading of Recommendations, Assessment, Development and Evaluation* (GRADE) was used to determine the level of certainty of the evidence (Atkins et al. 2004). This system has five domains: risk of bias, inconsistency, indirectness, imprecision, and publication bias. The overall quality was classified as high, moderate, low, or very low. The quality of all outcomes was determined by two independent reviewers (T. S. N. and R. O. L. P.), and any disagreements were resolved by a third reviewer (E. M.).

### Statistical analysis

This systematic review and meta-analysis followed the Cochrane Collaboration and the Preferred Reporting Items for Systematic Reviews and Meta-Analysis (*PRISMA*) statement guidelines (Page et al. 2021). *Review Manager Web* (The Cochrane Collaboration, 2023) was used for this data analysis (Review Manager Web (RevMan Web) 2023). The odds ratios (OR) with 95% confidence intervals were calculated, and the Mantel–Haenszel tool was used to compare intervention and control effects for categorical endpoints. A mean with a 95% confidence interval and inverse variance were used for continuous outcomes.

Cochran’s *Q* test and *I*^2^ statistics were used to assess the heterogeneity among studies. It was categorized as low (*I*^2^ = 0–40%), moderate (*I*^2^ = 30–60%), substantial (*I*^2^ = 50–90%), or considerable (*I*^2^ = 75–100%), according to the Cochrane Handbook guidelines (Higgins 2022). A random-effect model was chosen for all outcomes due to the risk of heterogeneity among groups. Publication bias was investigated by funnel plot analysis, and Egger’s linear regression was not used because fewer than 10 studies were included in the outcome. Sensitivity analysis by means of subgroup analysis was performed to identify potential causes of heterogeneity. The statistical significance of the research was set at *p* < *0.05*.

## Results

### Study selection and characteristics

**Fig. 1:**
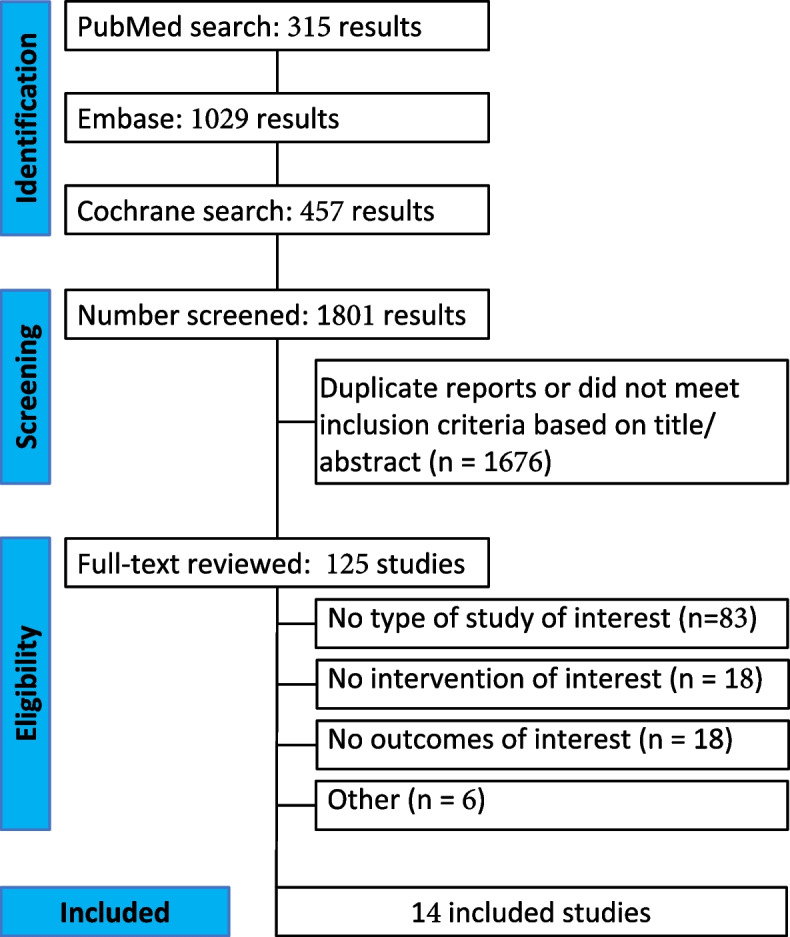
Initial search

As detailed in Fig. 1, the initial search returned 1801 studies. After an initial screening and title analysis, 1676 studies were removed due to ineligibility for being duplicates or previously retracted. The remaining 125 studies were thoroughly reviewed. Further, 111 studies were excluded based on the inclusion and exclusion criteria. Of these, 14 studies were conducted with 2143 patients from 11 randomized controlled trials (RCTs) and 3 nonrandomized studies, categorized into 2 cohorts and 1 matched case control. Eight-hundred and eighty-two (41%) received GLP-1RAs, and 1261 (59%) received control (placebo or insulin or no GLP-1RA). One RCT that met all inclusion criteria was excluded of the meta-analysis due to high risk of bias (confounding) and errors (Sindhvananda et al. 2023). Study characteristics are reported in (Table 1. Kaneko et al. 2018; Hulst et al. 2019; Besch et al. 2017; Besch et al. 2018; Holmberg et al. 2014; Kohl et al. 2014; Lipš et al. 2017; Makino et al. 2019; Polderman et al. 2018; Sokos et al. 2007; Kobori et al. 2023; Stark et al. 2022; Sherwin et al. 2023; Silveira et al. 2023; Hulst et al. 2020). A detailed summary of randomized controlled trials can be seen in Table S1. A detailed summary of observational studies can be seen in Table S2.

**Table 1:** Baseline characteristics of included studies

Study	Design	*n* (GLP-1RA/control)	*n* diabetics (%)	Type of procedure
Besch (2017)	RCT	104 (53/51)	22 (21.15)	Cardiac surgery
Besch (2018)	RCT	92 (49/43)	19 (20.65)	Cardiac surgery
Holmberg (2014)	RCT	42 (21/21)	10 (23.81)	Cardiac surgery
Hulst (2019)	RCT	261 (129/123)	42 (16.09)	Cardiac surgery
Hulst (2020)	RCT	261 (129/132)	42 (16.09)	Cardiac surgery
Kaneko (2018)	RCT	90 (49/41)	90 (100)	Noncardiac surgery
Kohl (2014)	RCT	77 (37/40)	11 (14.28)	Cardiac surgery
Lips (2017)	RCT	38 (19/19)	26 (68.42)	Cardiac surgery
Makino (2019)	RCT	70 (36/34)	70 (100)	Cardiac surgery
Polderman (2018)	RCT	97 (44/53)	97 (100)	Noncardiac surgery
Sokos (2007)	RCT	20 (10/10)	5 (25)	Cardiac surgery
Kobori (2023)	Case control	410 (205/205)	410 (100)	Endoscopy
Stark (2022)	Retrospective cohort	177 (59/118)	173 (97.74)	Endoscopy
Silveira (2023)	Retrospective cohort	404 (33/371)	38 (9.40)	Endoscopy

Abbreviations: *n* number of patients. *RCT* randomized controlled trials

### Perioperative implications in the use of GLP-1RAs when compared to control (placebo or insulin or no GLP1-RA)

#### Pre-procedural GI symptoms, RGC, and pulmonary aspiration

**Fig. 2:**
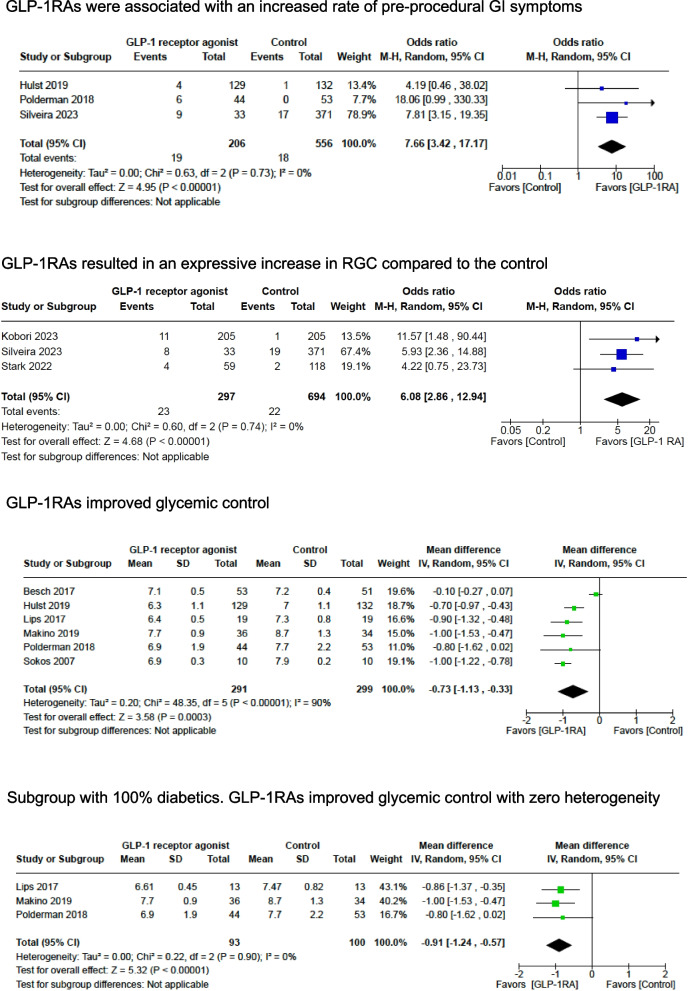
Study outcomes: GLP-1RAs were associated with an increased rate of pre-procedural GI symptoms, GLP-1RAs resulted in an expressive increase in RGC compared to the control, GLP-1RAs improved glycemic control, and subgroup with 100% diabetics and GLP-1RAs improved glycemic control with zero heterogeneity

Two randomized and one nonrandomized study assessed the incidence of pre-procedural GI symptoms in 762 patients undergoing cardiac and noncardiac surgeries and endoscopies. GLP-1RAs resulted in an increased rate of pre-procedural GI symptoms compared to control, with a significant difference in the pooled effect size (*OR* 7.66; 95% *CI* 3.42, 17.17; *p* < 0.00001; *I*^2^ = 0%) (Fig. 2) (Hulst et al. 2019; Polderman et al. 2018; Silveira et al. 2023).

Three nonrandomized studies showed a higher incidence of elevated RGC content in 1011 patients who used GLP-1RAs (*OR* 6.08; 95% *CI* 2.86, 12.94; *p* < 0.00001; *I*^2^ = 0%) despite adherence to current fasting recommendations (Fig. 2) (Kobori et al. 2023; Stark et al. 2022; Sherwin et al. 2023; Silveira et al. 2023). A summary of the characteristics of these studies can be seen in Table S2.

Silveira et al. revealed that patients taking semaglutide were five times more likely to have increased RGC during upper endoscopy procedures (*PR* 5.15; 95% *CI* 1.92, 12.92). This study reported one pulmonary aspiration case in a patient under deep sedation despite 12.4 h of fasting (Silveira et al. 2023).

Stark et al. presented that patients taking GLP-1RAs (exenatide or semaglutide) were four times more likely to have increased RGC during upper endoscopy than control (*OR* 4.22; 95% *CI* 0.75, 23.3) (Stark et al. 2022).

Kobori et al. demonstrated that patients taking GLP-1RAs (liraglutide, dulaglutide, or semaglutide) were 11 times more likely to have increased RGC during upper endoscopy than control (*OR* 11.57; 95% *CI* 1.48, 90.44) (Kobori et al. 2023).

#### Glycemic control and need for rescue insulin

The glycemic level during the perioperative period in cardiac and noncardiac surgeries was assessed by 6 randomized studies with 590 patients. GLP-1RA resulted in lower glycemic levels with a significant difference in the pooled effect size (*MD* − 0.73; 95% *CI* − 1.13, − 0.33; *p* = 0.0003; *I*^2^ = 90%) (Fig. 2) (Hulst et al. 2019; Besch et al. 2017; Lipš et al. 2017; Makino et al. 2019; Polderman et al. 2018; Sokos et al. 2007).

**Fig. 3:**
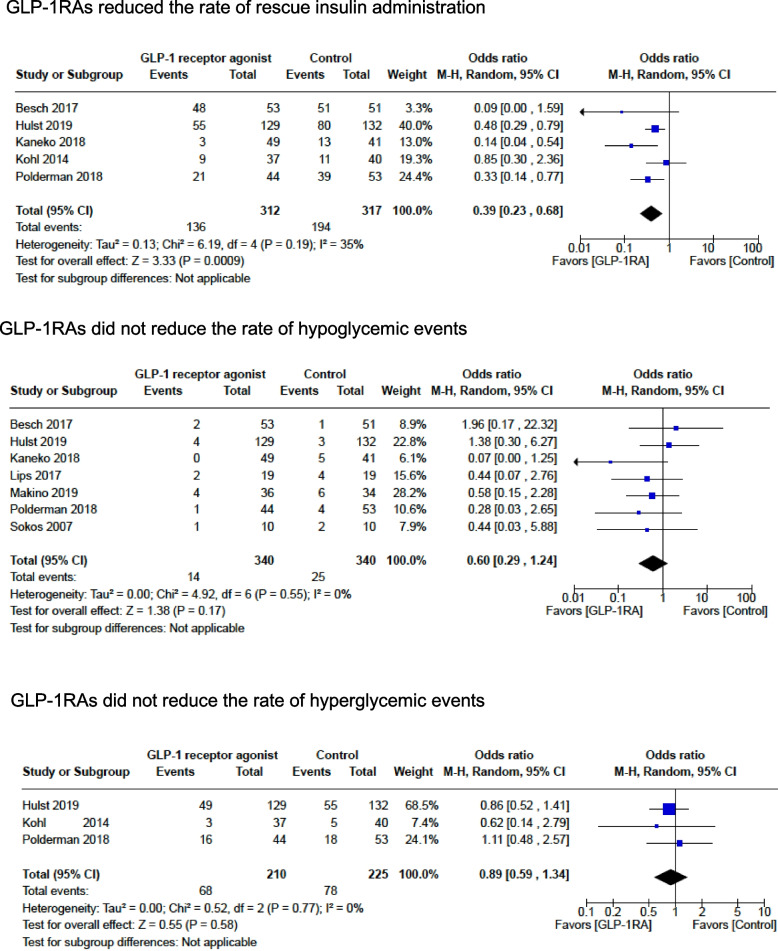
Study outcomes: GLP-1RAs reduced the rate of rescue insulin administration, GLP-1RAs did not reduce the rate of hypoglycemic events, and GLP-1RAs did not reduce the rate of hyperglycemic events

The rate of rescue insulin administration was assessed by 5 randomized studies with 629 patients. GLP-1RA use decreased the need for rescue insulin administration, with a significant difference in the pooled effect size (*OR* 0.39; 95% *CI* 0.23, 0.68; *p* = 0. 0009; *I*^2^ = 35%) (Fig. 3) (Kaneko et al. 2018; Hulst et al. 2019; Besch et al. 2017; Kohl et al. 2014; Polderman et al. 2018).

The rate of hypoglycemic events during the perioperative period was assessed by 7 randomized studies with 680 patients. GLP-1RA did not decrease the rate of hypoglycemic events in the pooled effect size (*OR* 0.60; 95% *CI* 0.29, 1.24; *p* = 0.17; *I*^2^ = 0%) (Fig. 3) (Kaneko et al. 2018; Hulst et al. 2019; Besch et al. 2017; Lipš et al. 2017; Makino et al. 2019; Polderman et al. 2018; Sokos et al. 2007).

The rate of hyperglycemic events during the perioperative period was assessed by 3 randomized studies with 435 patients. GLP-1RA did not reduce the rate of hyperglycemic events in the pooled effect size (*OR* 0.89; 95% *CI* 0.59, 1.34; *p* = 0.58; *I*^2^ = 0%) (Fig. 3) (Hulst et al. 2019; Kohl et al. 2014; Polderman et al. 2018).

#### Postoperative inotropic support

**Fig. 4:**
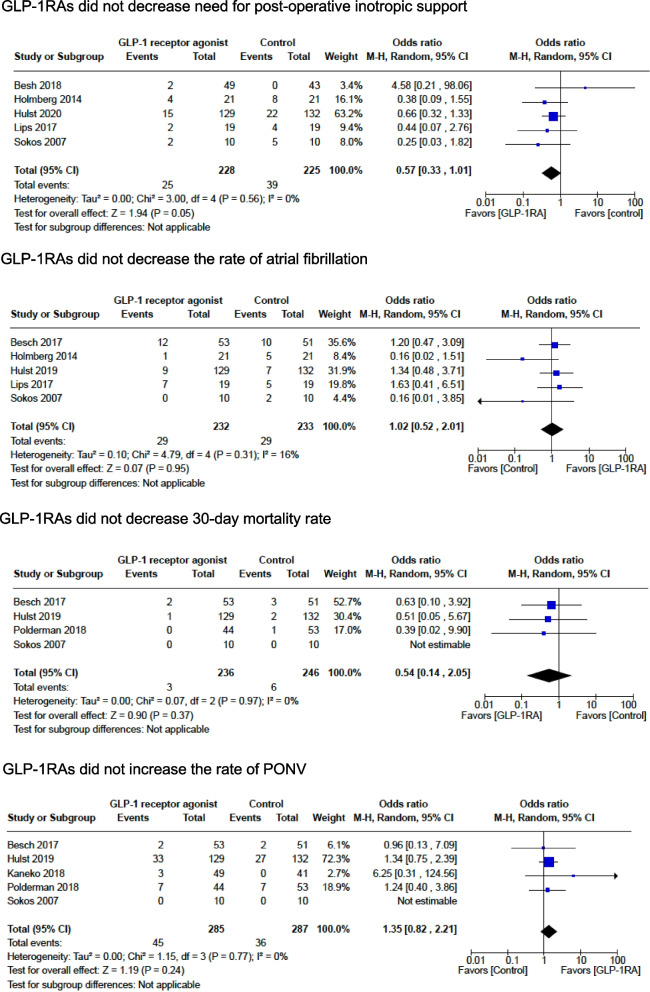
Study outcomes: GLP-1RAs did not decrease need for post-operative inotropic support, GLP-1RAs did not decrease the rate of atrial fibrillation, GLP-1RAs did not decrease 30-day mortality rate, and GLP-1RAs did not increase the rate of PONV

The rate of utilization of inotropic support after cardiac surgery was assessed by 5 randomized studies with 453 patients. GLP-1RA use did not result in a lower rate of inotropic support in the pooled effect size (*OR* 0.57; 95% *CI* 0.33, 1.01; *p* = 0.05; *I*^2^ = 0%) (Fig. 4) (Besch et al. 2018; Holmberg et al. 2014; Lipš et al. 2017; Sokos et al. 2007; Hulst et al. 2020).

#### Postoperative atrial fibrillation

The rate of atrial fibrillation in the first 30 days after cardiac surgery was assessed by 5 randomized studies in 465 patients. GLP-1 RA did not decrease the rate of atrial fibrillation in the pooled effect size (*OR* 1.02; 95% *CI* 0.52, 2.01; *p* = 0.95; *I*^2^ = 16%) (Fig. 4) (Hulst et al. 2019; Besch et al. 2017; Holmberg et al. 2014; Lipš et al. 2017; Sokos et al. 2007).

#### 30-day postoperative mortality rate

The 30-day mortality rate after cardiac and noncardiac surgery was assessed by 5 randomized studies in 482 patients. GLP-1RA did not decrease the 30-day mortality rate in the pooled effect size (*OR* 0.54; 95% *CI* 0.14, 2.05; *p* = 0.37; *I*^2^ = 0%) (Fig. 4) (Hulst et al. 2019; Besch et al. 2017; Polderman et al. 2018; Sokos et al. 2007).

#### Postoperative nausea and vomiting (PONV)

The rate of PONV was assessed by 5 randomized studies with 572 patients. GLP-1RA use did not increase the rate of PONV in the pooled effect size (*OR* 1.52; 95% *CI* 0.83, 2.81; *p* = 0.18; *I*^2^ = 13%) (Fig. 4) (Kaneko et al. 2018; Hulst et al. 2019; Besch et al. 2017; Polderman et al. 2018; Sokos et al. 2007).

### Quality assessment

Randomized studies were assessed through RoB 2 (Sterne et al. 2019), and the overall risk of bias was classified as low and some concerns (Fig. S1). Observational studies were assessed through ROBINS-I (Sterne et al. 2016), and due to the risk of confounding, they were classified as a moderate risk of bias (Fig. S1) (McGuinness and Higgins 2020). On funnel plot analysis, the studies occupied symmetrical distribution based on weight and converged toward the pooled effect as the weight increased, so there was no evidence of publication bias (Fig. S1) (Review Manager Web (RevMan Web) 2023). According to the GRADE system, the overall level of certainty of the evidence in this meta-analysis was high and moderate (Table 2).

**Table 2:** High and moderate overall level of certainty of the evidence in this meta-analysis

Outcomes	No. of participants (studies) Follow-up	Certainty of the evidence (GRADE)	Relative effect (95% *CI*)	Anticipated absolute effects	
				**Risk with control**	**Risk difference with GLP-1RA**
Preoperative gastrointestinal symptoms	762 (3 RCTs)	⨁⨁⨁⨁ High^a^	***OR* 7.66** (3.42 to 17.70)	32 per 1000	**172 more per 1000** (70 more to 340 more)
Insulin rescue administration	629 (5 RCTs)	⨁⨁⨁⨁ High	***OR* 0.39** (0.23 to 0.68)	612 per 1000	**231 fewer per 1000** (346 fewer to 94 fewer)
Glycemic level	590 (6 RCTs)	⨁⨁⨁⨁ High	-		*MD* **0.73 lower** (1.13 lower to 0.33 lower)
Hypoglycemic events	680 (7 RCTs)	⨁⨁⨁◯ Moderate^b^	***OR* 0.60** (0.29 to 1.24)	74 per 1000	**28 fewer per 1000** (51 fewer to 16 more)
Hyperglycemic events	435 (3 RCTs)	⨁⨁⨁⨁ High	***OR* 0.89** (0.59 to 1.34)	347 per 1000	**26 fewer per 1000** (108 fewer to 69 more)
Postoperative inotropic support assessed with the following: use of dobutamine, milrinone, and epinephrine	453 (5 RCTs)	⨁⨁⨁◯ Moderate^b^	***OR* 0.57** (0.33 to 1.01)	173 per 1000	**67 fewer per 1000** (109 fewer to 1 more)
Postoperative nausea/vomiting (PONV)	572 (5 RCTs)	⨁⨁⨁◯ Moderate^b^	***OR* 1.35** (0.82 to 2.21)	125 per 1000	**37 more per 1000** (20 fewer to 115 more)
Postoperative atrial fibrillation (A fib)	465 (5 RCTs)	⨁⨁⨁◯ Moderate^b^	***OR* 1.02** (0.52 to 2.01)	124 per 1000	**2 more per 1000** (56 fewer to 98 more)
30-day postoperative mortality rate	482 (4 RCTs)	⨁⨁⨁◯ Moderate^b^	***OR* 0.54** (0.14 to 2.05)	24 per 1000	**11 fewer per 1000** (21 fewer to 24 more)
Residual gastric content assessed with endoscopy	991 (3 nonrandomized studies)	⨁⨁⨁⨁ High^c^	***OR* 6.08** (2.86 to 12.94)	33 per 1000	**139 more per 1000** (56 more to 274 more)

*Patient or population*: patients undergoing anesthesia/sedation. *Setting*: surgical and nonsurgical procedures. *Intervention*: GLP-1RA. *Comparison*: control. *The risk in the intervention group (and its 95% confidence interval) is based on the assumed risk in the comparison group and the *relative effect* of the intervention (and its 95% CI). *CI*, confidence interval; *MD*, mean difference; *OR*, odds ratio. *GRADE Working Group grades of evidence*: *High certainty*, we are very confident that the true effect lies close to that of the estimate of the effect; *moderate certainty*, we are moderately confident in the effect estimate: the true effect is likely to be close to the estimate of the effect, but there is a possibility that it is substantially different; *low certainty*, our confidence in the effect estimate is limited: the true effect may be substantially different from the estimate of the effect; *very low certainty*, we have very little confidence in the effect estimate: the true effect is likely to be substantially different from the estimate of effect. Explanations: ^a^One observational study was included and was classified as moderate by ROBINS-I. ^b^Few events. ^c^Only observational studies and the risk of bias were classified as moderate by ROBINS-I

## Discussion

In this systematic review and meta-analysis of 14 studies with 2143 patients, GLP-1 RA was compared with insulin or placebo in adult patients undergoing cardiac and noncardiac surgeries or procedures with sedation/anesthesia. The main findings from the pooled population analysis were as follows: (1) GLP-1RAs were associated with an increased rate of pre-procedural GI symptoms (nausea, vomiting, dyspepsia, abdominal distension) compared to control, (2) GLP-1RA use resulted in an expressive increase in RGC compared to the control, (3) GLP-1RAs improved glycemic control and decreased the rate of rescue insulin administration compared to control, and (4) there was no significant difference between GLP-1RAs and control related to the rates of perioperative hypo or hyperglycemia, postoperative inotropic support, PONV, atrial fibrillation, and 30-day mortality.

Our results corroborate the concern that perioperative use of GLP-1RA might put patients at risk for pulmonary aspiration at induction of general anesthesia or sedation with an unprotected airway. This is further supported by several case reports (Table S3), which revealed solid gastric content via point-of-care ultrasound (POCUS), gastroscopy, or regurgitation despite proper fasting time according to current guidelines (Beam 2023; Fujino et al. 2023; Gulak and Murphy 2023; Klein and Hobai 2023; Wilson et al. 2023; Raven et al. 2023; Weber et al. 2023; Kittner et al. 2023; Queiroz et al. 2023). Sherwin et al. showed that in the group who used semaglutide, the rate of identification of solid content on gastric ultrasound was 70% when supine versus 10% in the control (*RR* 3.5, 95% *CI* 1.26–9.65, *p* = 0.02) and 90% when on lateral decubitus, versus 20% in the control group (*RR* 7.36, 95% *CI* 1.13, 47.7 *p* = 0.005) (Sherwin et al. 2023).

Gastric emptying delay, one of the desired effects of GLP-1RAs for weight loss and glycemic control, is achieved by inhibiting stomach peristalsis and augmenting pyloric contraction, which leads to a sensation of fullness and satiety (Aldawsari et al. 2023; Marroquin-Harris and Olesnicky 2023). Long-term, poorly controlled diabetics with autonomic dysfunction may be especially sensitive to this property of GLP-1RA (Marroquin-Harris and Olesnicky 2023; Joshi 2024).

In response to the concern of increased pulmonary aspiration risk in patients under GLP-1RA treatment, the American Society of Anesthesiologists (*ASA*) has released specific recommendations to guide patient care (Joshi et al. 2023). Although the discussion of these guidelines is beyond the scope of this article, it is worth mentioning that we have included the rate of GI symptoms associated GLP-1RAs in our meta-analysis, because the ASA considered the presence of severe GI symptoms preoperatively, a surrogate for increased RGC.

Furthermore, GLP-1RA pharmacokinetics is of paramount importance for the decision on when to discontinue the medication (Table S4) (Joshi et al. 2023; Quast et al. 2020). Ideally, one should wait at least five half-lives for total body clearance of the drug (Beam 2023). However, for long-acting GLP-1RAs, such as semaglutide, with a half-life of 1 week, that is not reasonable, as the patient would be deprived of the benefits of the medication for too long (Beam 2023; Sattar et al. 2021). On the other hand, with prolonged GLP-1RA use, there is evidence that delayed gastric emptying is reduced due to tolerance and tachyphylaxis (Holmberg et al. 2014; Umapathysivam et al. 2014; Halawi et al. 2017). Finally, there is not enough evidence to support recommendations on optimal drug discontinuation time and fasting time in patients using these drugs (Joshi et al. 2023; Klein and Hobai 2023; Marroquin-Harris and Olesnicky 2023).

Minimizing dysglycemia (glycemic variability, hypoglycemia, and hyperglycemia) is crucial to mitigate complications after cardiac and noncardiac surgery (Besch et al. 2019; Carlsson et al. 2023; Sim et al. 2018; Sim et al. 2015; Subramaniam et al. 2014; Sato et al. 2017; Frisch et al. 2010; Galway et al. 2021). Treatment with insulin, aiming for currently recommended targets, may also cause hypoglycemia (Sreedharan et al. 2023). For this reason, GLP-1RAs have risen as alternative therapies, with the potential to improve glycemic control and minimize the need for rescue insulin administration (Kaneko et al. 2018; Hulst et al. 2019; Lipš et al. 2017; Polderman et al. 2018). Our study supports findings from previous studies, although we did not investigate glucose variability due to the scarcity of studies reporting this parameter. One important remark is that in this meta-analysis, we observed high heterogeneity associated with the investigated glycemic control outcomes. It resulted from different proportions of diabetics among participants of the studies. When the glycemic level outcome was investigated through a subgroup analysis consisting of 100% diabetics, the heterogeneity level dropped to zero.

Animal and clinical studies support the existence of cardioprotective effects of GLP-1RAs (Hulst et al. 2020; Sattar et al. 2021). Potential mechanisms include improved efficiency of myocardial glucose utilization, decreased systemic and pulmonary vascular resistance, activation of ischemic preconditioning pathways, chronotropism, improvement of systolic and diastolic function, among others (Aravindhan et al. 2015; Kim et al. 2013; Ravassa et al. 2012; Sheikh 2013). In the surgical setting, the cardioprotective effects of GLP-1RAs are yet to be demonstrated, with very modest results compared to animal and nonsurgical studies (Besch et al. 2018; Lipš et al. 2017). Given the scarcity of data, we explored the cardioprotective effects of these drugs indirectly by measuring the rate of inotropic support use in the postoperative period, rate of atrial fibrillation, and 30-day mortality. However, one caveat is that most available studies consist largely of normal ejection fraction coronary artery bypass grafts procedures, which reported low rates of inotropic support use in general: 11% in the GLP-1RA and 17% in the control group (Hulst et al. 2019; Besch et al. 2017; Besch et al. 2018; Holmberg et al. 2014; Lipš et al. 2017; Makino et al. 2019; Sokos et al. 2007). Therefore, we understand that this dichotomous (qualitative) analysis of inotropic use is a very crude way of investigation that could have missed subtle beneficial effects of GLP-1RAs.

Postoperative GI function is another concern for patients utilizing GLP-1RAs that is largely unexplored. In a nonsurgical population-based cohort that included 25,617 patients, Faillie et al. reported that patients receiving GLP-1RAs had an increased risk of intestinal obstruction compared to SGLT-2 inhibitors (*HR* 3.48; 95% *CI* 1.79, 6.79) (Faillie et al. 2022). Given the properties of these medications, one might speculate on the increased risk of the rate of PONV and postoperative ileus associated with its use, particularly after GI surgery (Camilleri and Lupianez-Merly 2023; Horowitz et al. 2017). We explored this risk by analyzing the rate of PONV, as we could not find studies reporting the rate of postoperative ileus.

### Strengths and limitations

The major strength of this study was reviewing the existing literature on the risks and benefits of using GLP-1RAs in the surgical setting and procedures under sedation/anesthesia from the perioperative physician perspective. To our knowledge, this is the first meta-analysis investigating pre-procedural RGC in patients using GLP-1RAs. However, this study has some limitations. First, there were no randomized studies that analyzed fasting RGC in patients using GLP-1RAs in the surgical setting. Consequently, our results related to RGC were based on observational studies, which may incur confounding. Therefore, optimal preoperative fasting time in this population remains unknown. Second, there was only a small number of randomized studies to serve as a basis for the exploration of perioperative glycemic control and postoperative cardiovascular and GI outcomes, which may limit the generalizability of our results.

## Conclusion

Compared to control, pre-procedural GLP-1RA was associated with an increased rate of GI symptoms and with elevated residual gastric content despite adherence to fasting guidelines. GLP-1RAs improved glycemic control and decreased the rate of rescue insulin administration. There was no significant difference in the rates of perioperative hypo or hyperglycemia, postoperative inotropic support use, PONV, atrial fibrillation, and 30-day mortality. Given the relatively small number of trials analyzed, additional studies are required to establish the optimal timing of GLP-1RA discontinuation before surgery and further explore its impact on perioperative glycemic control and postoperative cardiovascular and GI outcomes.

### Supplementary Information

Supplementary Material 1. Supplementary figure: Figure S1. Quality assessment.

Supplementary Material 2. Supplementary tables: Table S1. Summary of included randomized controlled trials. Table S2. summary of characteristics of observational studies. Table S3. Case reports on increased residual gastric content and/or pulmonary aspiration related to anesthesia. Table S4. GLP-1RA pharmacokinetics

## Data Availability

No datasets were generated or analysed during the current study.

## Abbreviations

PR: Prevalence ratio
OD: Odds ratio
CI: Confidence interval
MD: Mean difference
PRISMA: Preferred Reporting Items for Systematic Reviews and Meta-Analysis
GRADE: Grades of Recommendation, Assessment, Development, and Evaluation
RCT: Randomized controlled trial(s)
PONV: Postoperative nausea and vomiting
GLP-1RAs: Glucagon-like peptide-1 receptor agonists
POCUS: Point-of-care ultrasound
RGC: Residual gastric content
GI: Gastrointestinal
