# Supplementary material for: The impact of glucagon-like peptide-1 receptor agonists in the patients undergoing anesthesia or sedation: systematic review and meta-analysis
Source: Perioper Med (Lond). 2024 Jul 22;13:78. doi: 10.1186/s13741-024-00439-y (PMC11264430; doi:10.1186/s13741-024-00439-y)

Figure S1. Quality assessment

The risk of bias of randomized studies was classified as low and some concerns

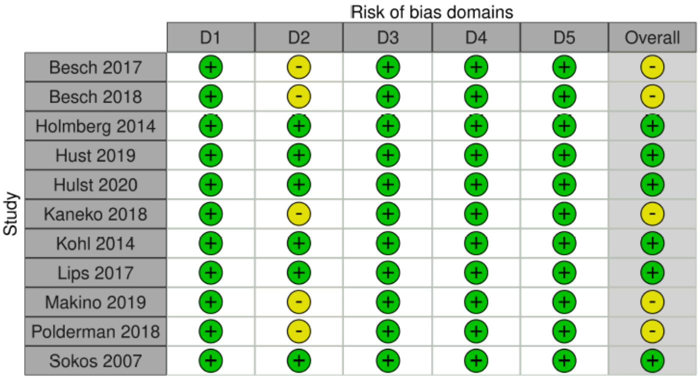

Domains:
D1: Bias arising from the randomization process.
D2: Bias due to deviations from intended intervention.
D3: Bias due to missing outcome data.
D4: Bias in measurement of the outcome.
D5: Bias in selection of the reported result.

Judgement
- Some concerns
+ Low

The risk of bias of non-randomized studies was classified moderate

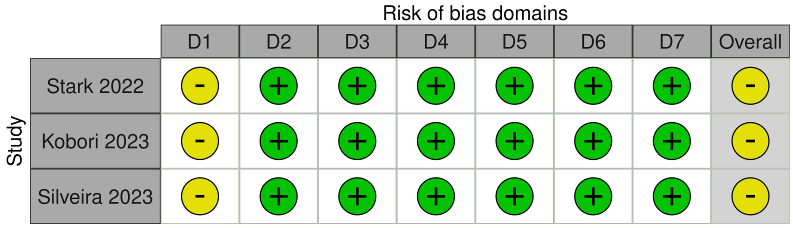

Domains:
D1: Bias due to confounding.
D2: Bias due to selection of participants.
D3: Bias in classification of interventions.
D4: Bias due to deviations from intended interventions.
D5: Bias due to missing data.
D6: Bias in measurement of outcomes.
D7: Bias in selection of the reported result.

Judgement
- Moderate
+ Low

The funnel plot for pre-procedural GI symptoms (left) and residual gastric content (right) demonstrated no evidence of publication bias

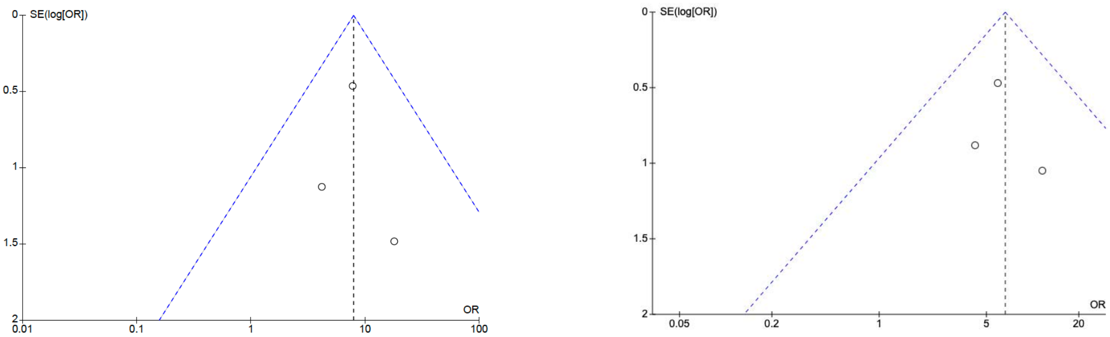

Supplement: Supplementary file 1 — Supplementary Material 1. Supplementary figure: Figure S1. Quality assessment. [file 13741_2024_439_MOESM1_ESM.pdf]
