# Supplementary material for: The impact of glucagon-like peptide-1 receptor agonists in the patients undergoing anesthesia or sedation: systematic review and meta-analysis
Source: Perioper Med (Lond). 2024 Jul 22;13:78. doi: 10.1186/s13741-024-00439-y (PMC11264430; doi:10.1186/s13741-024-00439-y)
Supplement: Supplementary file 2 — Supplementary Material 2. Supplementary tables: Table S1. Summary of included randomized controlled trials. Table S2. summary of characteristics of observational studies. Table S3. Case reports on increased residual gastric content and/or pulmonary aspiration related to anesthesia. Table S4. GLP-1RA pharmacokinetics [file 13741_2024_439_MOESM2_ESM.zip › Table S1.docx]

Table S1. Summary of included randomized controlled trials

| **Study** | **Intervention** | **Intervention timing** | **Control** | **Number of patients (GLP-1RA/Control)** | **Type of surgery** | **Outcomes of interest** |
| --- | --- | --- | --- | --- | --- | --- |
| Besch 2017 | Exenatide  I.V. infusion | Started with the first blood glucose value > 7.7 mmol/L and continued for 48 h after incision | Insulin I.V. infusion | 104 (53/51) | CABG | Glycemic level, hypoglycemic events, rescue insulin administration, Atrial fibrillation, PONV, mortality |
| Besch 2018 | Exenatide  I.V. infusion | During the first 48 h after incision | Insulin I.V. infusion | 92 (49/43) | CABG | Postoperative inotropic support |
| Holmberg  2014 | Exenatide  I.V. infusion | Started 30 min before aortic cross-clamp and continued for 6h | Standard of care (not detailed) | 42 (21/21) | CABG, AVR, AA  Valve replacement  Myxoma | Postoperative inotropic support, Atrial fibrillation |
| Hulst 2019 | Liraglutide  subcutaneous | Evening before surgery and after anesthesia induction | Placebo (plus I.V. bolus) | 261 (129/132) | CABG | Pre-procedure GI symptoms, glycemic level, rescue insulin administration, hypoglycemic and hyperglycemic events  Atrial fibrillation, PONV, mortality |
| Hulst 2020 | Liraglutide  subcutaneous | Evening before surgery and after anesthesia induction | Placebo (plus I.V. bolus) | 261 (129/132) | CABG | Postoperative inotropic support |
| Kohl 2014 | GLP-1RA (7-36) amide I.V infusion | Started at anesthesia induction and stopped at the end of surgery | Placebo (plus insulin I.V. infusion) | 77 (37/40) | CABG  Valvuloplasty  Valve replacement | Hyperglycemic events, rescue insulin administration |
| Lips 2017 | Exenatide  I.V. infusion | Started 12 h before surgery and continued for 72h | Placebo (plus insulin I.V. infusion) | 38 (19/19) | CABG  Valvuloplasty | Glycemic level, hypoglycemic events, atrial fibrillation, postoperative inotropic support |
| Makino 2019 | Liraglutide  I.V. infusion | Perioperative period (not specified) | Insulin I.V. infusion | 70 (36/34) | CABG  Valve replacement | Glycemic events, hypoglycemic events |
| Sokos 2007 | Not specified GLP-1RA I.V. infusion | Started 12 h before surgery and continued for 72 h | Placebo (plus insulin I.V. infusion) | 20 (10/10) | CABG | Glycemic level, hypoglycemic events, postoperative inotropic support, Atrial fibrillation, PONV, mortality |
| Polderman 2018 | Liraglutide  subcutaneous | Evening before surgery and morning of surgery | Insulin I.V. infusion | 97 (44/53) | Noncardiac surgeries | Pre-procedure GI symptoms, glycemic level, rescue insulin administration, hypoglycemic and hyperglycemic events  PONV, mortality |
| Kaneko 2018 | Liraglutide  subcutaneous | Daily dosage for 7 or more days before surgery, continued for 48 h after surgery | Insulin I.V. infusion | 90 (49/41) | Noncardiac surgeries | Hypoglycemic events, rescue insulin administration, PONV |
