# Supplementary material for: The impact of glucagon-like peptide-1 receptor agonists in the patients undergoing anesthesia or sedation: systematic review and meta-analysis
Source: Perioper Med (Lond). 2024 Jul 22;13:78. doi: 10.1186/s13741-024-00439-y (PMC11264430; doi:10.1186/s13741-024-00439-y)
Supplement: Supplementary file 2 — Supplementary Material 2. Supplementary tables: Table S1. Summary of included randomized controlled trials. Table S2. summary of characteristics of observational studies. Table S3. Case reports on increased residual gastric content and/or pulmonary aspiration related to anesthesia. Table S4. GLP-1RA pharmacokinetics [file 13741_2024_439_MOESM2_ESM.zip › Table S2.docx]

Table S2. summary of characteristics of observational studies

| Study | Design | GLP-1RA | Control | *n* (GLP-1RA/control | Type of procedure | Outcomes of interest |
| --- | --- | --- | --- | --- | --- | --- |
| Kobori, 2023 | Matched pair case-control | Dulaglutide Liraglutide Semaglutide | No GLP-1RA | 410 (205/205) | Endoscopy | Residual gastric content (increased vs. not increased) |
| Silveira, 2023 | Retrospective cohort | Semaglutide | No GLP-1RA | 404 (33/371) | Endoscopy | Gastrointestinal symptoms, residual gastric content (increased vs. not increased) |
| Stark, 2022 | Matched control retrospective cohort | Dulaglutide Exenatide Liraglutide Semaglutide | No GLP-1RA | 177 (59/118) | Endoscopy | Residual gastric content. (increased vs. not increased) |
