# Supplementary material for: The impact of glucagon-like peptide-1 receptor agonists in the patients undergoing anesthesia or sedation: systematic review and meta-analysis
Source: Perioper Med (Lond). 2024 Jul 22;13:78. doi: 10.1186/s13741-024-00439-y (PMC11264430; doi:10.1186/s13741-024-00439-y)
Supplement: Supplementary file 2 — Supplementary Material 2. Supplementary tables: Table S1. Summary of included randomized controlled trials. Table S2. summary of characteristics of observational studies. Table S3. Case reports on increased residual gastric content and/or pulmonary aspiration related to anesthesia. Table S4. GLP-1RA pharmacokinetics [file 13741_2024_439_MOESM2_ESM.zip › Table S3.docx]

Table S3. Case reports on increased residual gastric content and/or pulmonary aspiration related to anesthesia

| **Author** | **Event** | **GLP-1 RA** | **Fasting for solids (h)** | **Procedure** |
| --- | --- | --- | --- | --- |
| Beam, 2023 | Residual gastric content | Semaglutide | > 18 | Gastric ultrasound |
| Beam, 2023 | Residual gastric content | Tizerpatide | > 8 | Hysterectomy |
| Fugino, 2023 | Residual gastric content | Semaglutide | > 10 | Upper endoscopy |
| Gulak, 2023 | Regurgitation of gastric contents | Semaglutide | 20 | Lumpectomy |
| Klein, 2023 | Pulmonary aspiration | Semaglutide | 18 | Upper endoscopy |
| Wilson, 2023 | Regurgitation of gastric content | Dulaglutide | 10 | Foot arthrodesis |
| Wilson, 2023 | Regurgitation + Residual gastric content | Semaglutide | 16 | Thyroidectomy |
| Raven, 2023 | Residual gastric content | Semaglutide | 13 | Gastroscopy |
| Raven, 2023 | Residual gastric content | Liraglutide | 10 | Gastroscopy |
| Weber, 2023 | Regurgitation of gastric content + Residual gastric content | Tizerpatide | > 8 | Hysteroscopy |
| Kittner, 2023 | Residual gastric content | Semaglutide | 11 | Total knee replacement |
| Kittner, 2023 | Residual gastric content | Semaglutide | 10 | Knee arthroscopy |
| Kittner, 2023 | Residual gastric content | Semaglutide | 14 | Knee arthroplasty |
| Queiroz, 2023 | Residual gastric content | Semaglutide | 9 | Renal nodule ablation |
