# Supplementary material for: The impact of glucagon-like peptide-1 receptor agonists in the patients undergoing anesthesia or sedation: systematic review and meta-analysis
Source: Perioper Med (Lond). 2024 Jul 22;13:78. doi: 10.1186/s13741-024-00439-y (PMC11264430; doi:10.1186/s13741-024-00439-y)
Supplement: Supplementary file 2 — Supplementary Material 2. Supplementary tables: Table S1. Summary of included randomized controlled trials. Table S2. summary of characteristics of observational studies. Table S3. Case reports on increased residual gastric content and/or pulmonary aspiration related to anesthesia. Table S4. GLP-1RA pharmacokinetics [file 13741_2024_439_MOESM2_ESM.zip › Table S4.docx]

Table S4. GLP-1RA pharmacokinetics.

|  | **Generic name** | **Brand name** | **Half-life** | **Clearance** |
| --- | --- | --- | --- | --- |
| **GLP-1RA**  **Short-acting** | Exenatide b.i.d | Byetta, Bydureon | 3 hours | Renal |
|  | Lixisenatide | Adlyxin | 3 hours | Renal |
| **GLP-1RA**  **Long-acting** | Dulaglutide | Trulicity | 4.5 days | Renal |
|  | Liraglutide | Saxenda, Victoza | 12.5 hours | Renal |
|  | Albiglutide | Tanzeum | 5 days | Renal |
|  | Semaglutide | Ozempic, Wegovy, Rybesius | 7 days | Renal |
| **GLP-1RA/GIP Agonist** | Tizerpatide | Mounjaro | 5 days | Renal |
